# Activated microglia and neuroinflammation as a pathogenic mechanism in Leigh syndrome

**DOI:** 10.3389/fnins.2022.1068498

**Published:** 2023-01-18

**Authors:** Nastaran Daneshgar, Mariah R. Leidinger, Stephanie Le, Marco Hefti, Alessandro Prigione, Dao-Fu Dai

**Affiliations:** ^1^Department of Pathology, Carver College of Medicine, University of Iowa, Iowa City, IA, United States; ^2^Department of General Pediatrics, Neonatology and Pediatric Cardiology, Medical Faculty, Heinrich Heine University, Düsseldorf, Germany

**Keywords:** Leigh syndrome, neuroinflammation, microglia, Ndufs4, brain organoid, Pexidartinib

## Abstract

Neuroinflammation is one of the main mechanisms leading to neuronal death and dysfunction in neurodegenerative diseases. The role of microglia as primary mediators of inflammation is unclear in Leigh syndrome (LS) patients. This study aims to elucidate the role of microglia in LS progression by a detailed multipronged analysis of LS neuropathology, mouse and human induced pluripotent stem cells models of Leigh syndrome. We described brain pathology in three cases of Leigh syndrome and performed immunohistochemical staining of autopsy brain of LS patients. We used mouse model of LS (Ndufs4^−/−^) to study the effect of microglial partial ablation using pharmacologic approach. Genetically modified human induced pluripotent stem cell (iPS) derived neurons and brain organoid with Ndufs4 mutation were used to investigate the neuroinflammation in LS. We reported a novel observation of marked increased in Iba1+ cells with features of activated microglia, in various parts of brain in postmortem neuropathological examinations of three Leigh syndrome patients. Using an Ndufs4^−/−^ mouse model for Leigh syndrome, we showed that partial ablation of microglia by Pexidartinib initiated at the symptom onset improved neurological function and significantly extended lifespan. Ndufs4 mutant LS brain organoid had elevated NLRP3 and IL6 pro-inflammatory pathways. Ndufs4-mutant LS iPSC neurons were more susceptible to glutamate excitotoxicity, which was further potentiated by IL-6. Our findings of LS human brain pathology, Ndufs4-deficient mouse and iPSC models of LS suggest a critical role of activated microglia in the progression of LS encephalopathy. This study suggests a potential clinical application of microglial ablation and immunosuppression during the active phase of Leigh syndrome.

## Key points

- Substantial increase in Iba1+ cells with features of activated microglia was observed in multiple regions of Leigh syndrome patients' brain, in association with neuronal loss.
- Microglial partial ablation in Leigh syndrome mouse model increased survival and ameliorated neurological deficit and neuropathology.
- Modeling by human induced pluripotent stem cells (iPS) derived neurons and brain organoid showed that Ndufs4-deficient iPS-neurons are more susceptible to glutamate excitotoxicity; Ndufs4-mutant iPS-brain organoid displays activated inflammatory pathways.
- We propose that suppressing neuroinflammation, either by microglial ablation or other immunosuppressants, may have a therapeutic potential in the management of Leigh syndrome patients, particularly during the active phase of neuroinflammation.

## Introduction

Leigh syndrome (LS) is a severe mitochondrial encephalomyopathy that usually becomes apparent in the first year of life, characterized by progressive loss of mental and movement abilities resulting in early mortality. The most common mutations of LS involved mitochondrial respiratory chain complex subunits or complex assembly proteins, particularly complex I, which account for ~33% of LS cases (Ruhoy and Saneto, 2014). NDUFS4 (NADH dehydrogenase [ubiquinone] iron-sulfur protein 4) is one of the 45 subunits of complex I that causes LS in humans. Leigh syndrome, also called subacute necrotizing encephalopathy, display neuropathological lesions characterized by vacuolation of the neuropil associated with demyelination, gliosis, and vascular proliferation, with milder degree of neuronal degeneration and depletion. The lesion topography is characterized by bilateral involvement of the brainstem, basal ganglia and cerebellum (Lake et al., 2015).

Neuroinflammation is proposed as one of the mechanisms leading to neuronal death and dysfunction in neurodegenerative diseases, such as Alzheimer's Disease (AD). Microglia comprise 5–20% of brain cells and are primary mediators of inflammation. Microglia maintain CNS homeostasis through interacting with neurons and other glial cells (Nimmerjahn et al., 2005). This is either through contact-independent mechanism such as releasing neurotrophic factors (Ueno et al., 2013), contact-dependent mechanism or complement-mediated manner (Schafer et al., 2012). Adult microglia have small cell bodies and multiple ramified processes which become activated upon any CNS insults which make them lose their processes and become ameboid-like (Davalos et al., 2005). These activated microglia are attracted to the site of insults, in order to engulf pathogens or cellular debris. Increases in the expression of markers of microglia, the main immune cells within the brain, have been extensively reported in brains from patients with AD (Hopperton et al., 2018). However, the role of microglia in mitochondrial encephalopathy is underexplored.

In this study, we characterized neuropathological features of three LS patients and reported novel finding of microgliosis and activated microglia in several regions of LS brains. To investigate the role of microglia in the pathogenesis of Leigh syndrome, we partially ablated microglia using Pexidartinib (Elmore et al., 2014) and demonstrated improved survival, in association with attenuation of IL6 increase in LS mice. The role of neuroinflammation in this scenario was confirmed using human iPSC derived neurons and brain organoid.

## Results

**Figure 1:**
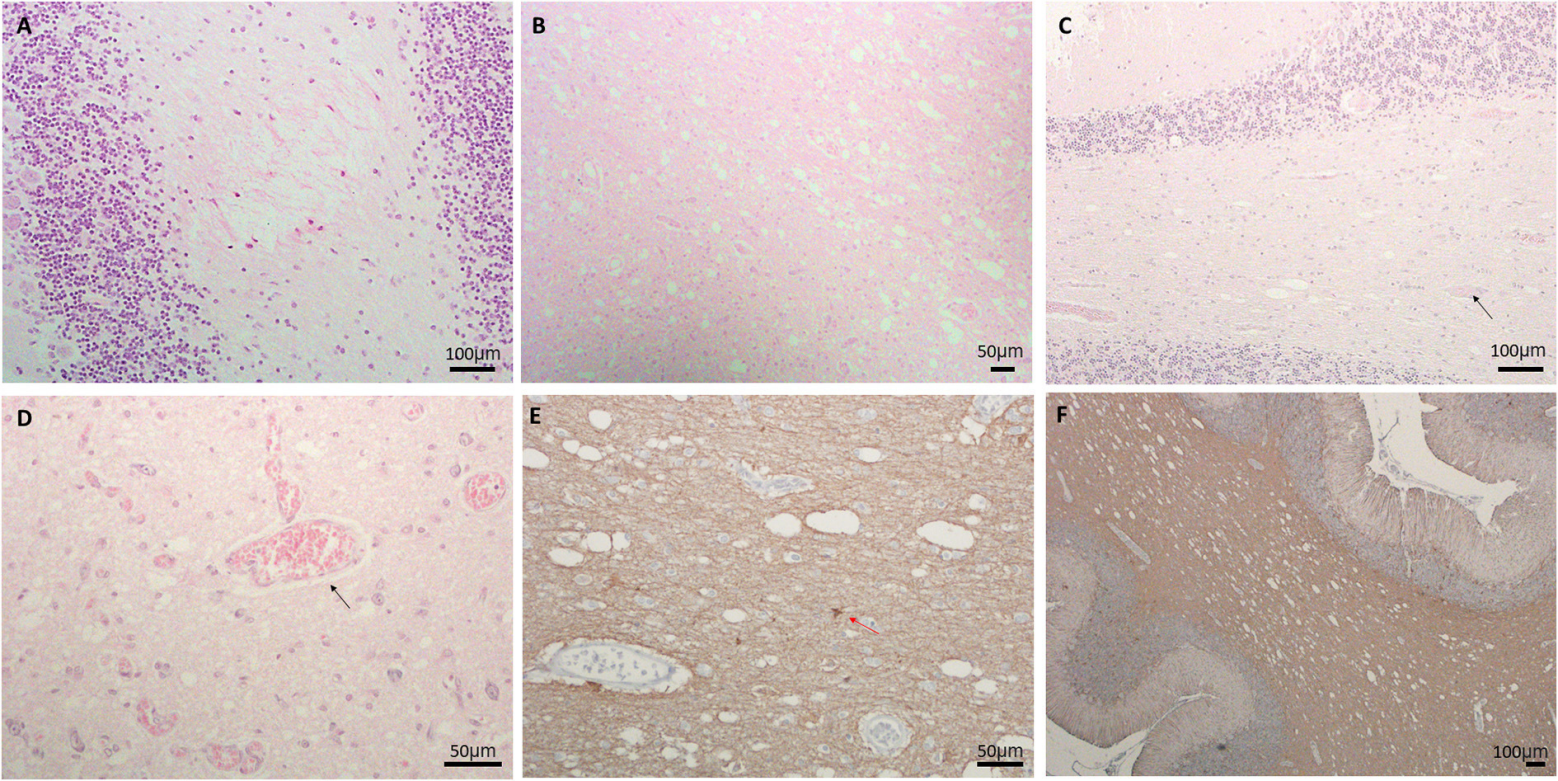
**(A)** Neuropil vacuolation in the cerebellar white matter in LS#l, **(B)** extensive vacuolation, gliosis, and vascular proliferation in pons of LS#2, **(C, D)** vacuolation, vessels (marked by black arrow) and decreased Purkinje cells in **(C)** LS#3 and **(D)** higher magnification of LS#2 showing scattered vessels (black arrow); H&E staining for **(A–D)**. **(E, F)** IHC of GFAP showing extensive gliosis with obvious neuropils vacuolation in LS#2 **(F)** high-magnification shows numerous vessels and reactive astrocytes in LS#3 [red arrow, **(E)**].

Here we report the clinical and neuropathological features from three LS patients (Table 1). The first case (LS-1) was a 14-month-old male presenting with lactic acidosis and encephalopathy. Gross examination of his brain showed ventriculomegaly, without obvious foci of necrosis, hemorrhage, or other architectural anomalies. Microscopic examination revealed prominent neuropil vacuolation in frontotemporal cortex, with reduced number of neurons, prominent gliosis, and increased capillaries (vascular proliferation). No evidence of storage disease was observed. In the cerebellum, there was enlarged dentate nuclei and slightly decreased Purkinje cells, associated with gliosis and vacuolation (Figure 1A). There was prominent capillary pattern and gliosis around the third ventricles and mamillary body, which is a characteristic feature of LS. The second case (LS-2) was an 8-month-old male with positive family history of LS and laboratory confirmation of decreased complex I and complex III activity, presenting with developmental delay. Neuropathological examination showed presence of necrotic lesions in the brainstem, with extensive vacuolation of white matters in the brainstem (Figure 1B), cerebellum and deeper portions of the cerebrum. The cerebellar cortex had well-developed internal granular and molecular cell layers. There seemed to be fewer Purkinje cells than expected, suggesting a Purkinje cell loss. The remaining Purkinje cells were without evidence of ischemic changes, inclusions, or storage disease. The brainstem showed multiple foci of necrotic lesions involving substantia nigra, midbrain tegmentum, base of pons, and tegmentum of the medulla. The most prominent findings were neuropil degeneration with vacuolation, proliferation of capillaries and gliosis. The third case (LS-3) was a 3-year-old with multiple necrotic foci in the striatum, dentate nuclei and cerebellar cortex, and brainstem, spanning from midbrain, pons to inferior olives and medulla. There was mild bilateral ventricular dilatation. The pathologic findings were characterized by moderate to severe gliosis, numerous capillaries (neovascularization), vacuolation and some degrees of neuronal loss. Purkinje cell loss with axonal swelling were also observed (Figures 1C, D). Extensive gliosis with evident neuropils vacuolation were shown using Glial fibrillary acidic protein (GFAP) staining (Figures 1E, F).

**Table 1:** Summary of clinical and pathological feature of three Leigh syndrome patients.

**Cases**	**Clinical data**	**Neuropathological findings**
LS #1	14-month male • Lactic acidosis • Encephalopathy	•Frontal/temporal regions: vacuolation, gliosis, and neovascularization • Cerebellum: enlarged dentate nuclei and Purkinje cells with slightly decreased neuron number • Capillary/neovascular accentuation around the third ventricle
LS #2	236-day-old male • Positive family history (sibling died at 8-month-old due to Leigh Disease) • Partial deficiency of Rotenone-sensitive complex I activity and decreased complex III	• Necrotic lesions in brainstem • Vacuolation of white matters in the brainstem, cerebellum and cerebral cortex • Relatively preserved cerebral cortex with a few scattered ischemic neurons
LS #3	3-year-old male • Decreased complex IV	• Vasculo-necrotic foci most prominently affecting striatum, dentate nuclei and cerebellar cortex, and brainstem, spanning from midbrain to medulla at the level of inferior olives. • Moderate to severe gliosis, capillary prominence, and vacuolization • Some degrees of neuronal loss, including loss of Purkinje cell, with axonal swelling.

H&E staining showing.

**Figure 2:**
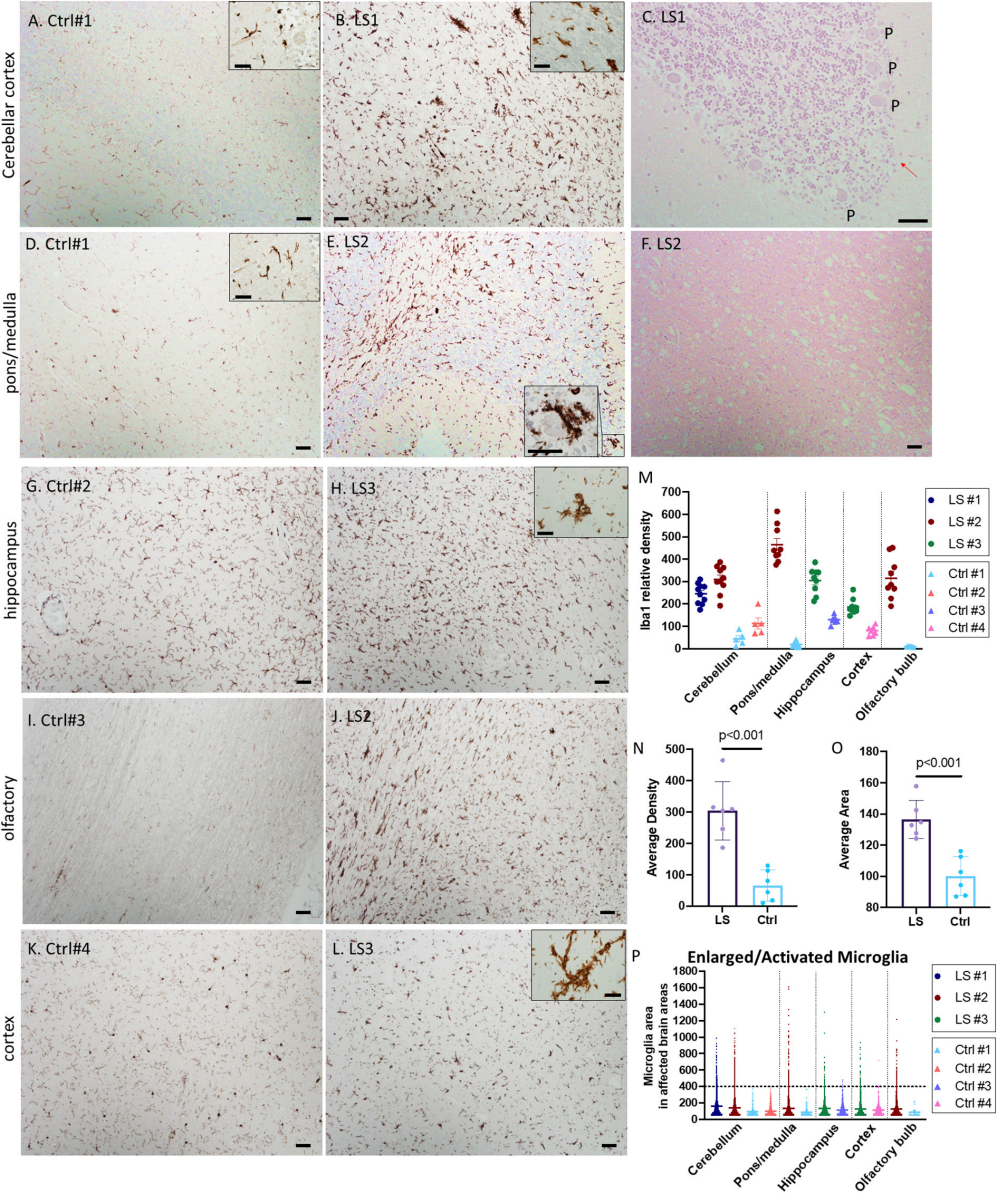
**(A–F)** Representative IHC for Iba1 and corresponding H&E staining of Leigh syndrome cerebellum and pons/medulla. Compared with controls **(A, D)**, there is substantial increase in microglia indicated by Iba1 staining in the LS cerebellar pons **(B)** and cortex **(E)**. High magnification of Iba1+ activated microglia's cytoplasm partially surrounding a Purkinje neuron (**E**, inset) and the corresponding area displayed the loss of Purkinje (P) neurons [**(C)**, red arrow]. **(F)** The corresponding H&E staining of LS pons from **(E)** showed neuropil vacuolization. **(G–L)** Representative IHC for Iba1 of hippocampus, olfactory and cerebral cortex in control brains (left panels) vs. brains from Leigh syndrome patients (LS2,3, right panels). Compared with controls, there is substantial increase in microglia marked by Iba1 staining. Multiple affected regions of brains from three LS patients and controls were analyzed. **(M)** Relative densities of Iba1+ cells in various affected brain regions. **(N)** Analysis of all data from multiple regions of LS vs. controls. **(O, P)** Cell size of Iba1+ microglia showed several enlarged activated microglia in LS brains; 5–10 images from multiple regions of brains from three LS patients and four controls were analyzed. Scale bar: 50 μm. Inset scale: 25 μm.

White matter vacuolation, capillary proliferation and gliosis were common features in all three cases. We performed immunohistochemistry (IHC) for ionized calcium binding molecule 1 (Iba1) to label microglia. As shown in Figure 2, there was a substantial increase in microglial number (>3-fold) and cell size in multiple regions involved in LS pathology, more prominently in the cerebellum (Figures 2A–C), lower pons/upper medulla (Figures 2D–F). Increase in activated microglia was also observed in hippocampus, cortex, and olfactory bulb (Figures 2G–L). Interestingly, many of the activated microglia were in close proximity or in direct contact with neurons in the granular layers and Purkinje neurons (Figure 2E, inset), suggesting neuronophagia, a phenomenon commonly believed to be characteristic of viral or autoimmune encephalitis but not previously described in LS. The activated microglia immediately adjacent to Purkinje neurons were associated with loss of Purkinje neurons in the cerebellum (arrow in Figure 2C, H&E). Quantitative analysis of multiple images of three LS cases shows ~2–4-fold increase in the relative density of microglia, Iba1 positive cells, in multiple affected brain regions, more prominently in pons/medulla and cerebellum (Figures 2M, N). In addition, many of these microglia were enlarged, with an average of ~30% increase in the size of microglia, predominantly due to enlarged soma (Figures 2O, P), suggesting increases in both number and activity of microglia in LS brains.

**Figure 3:**
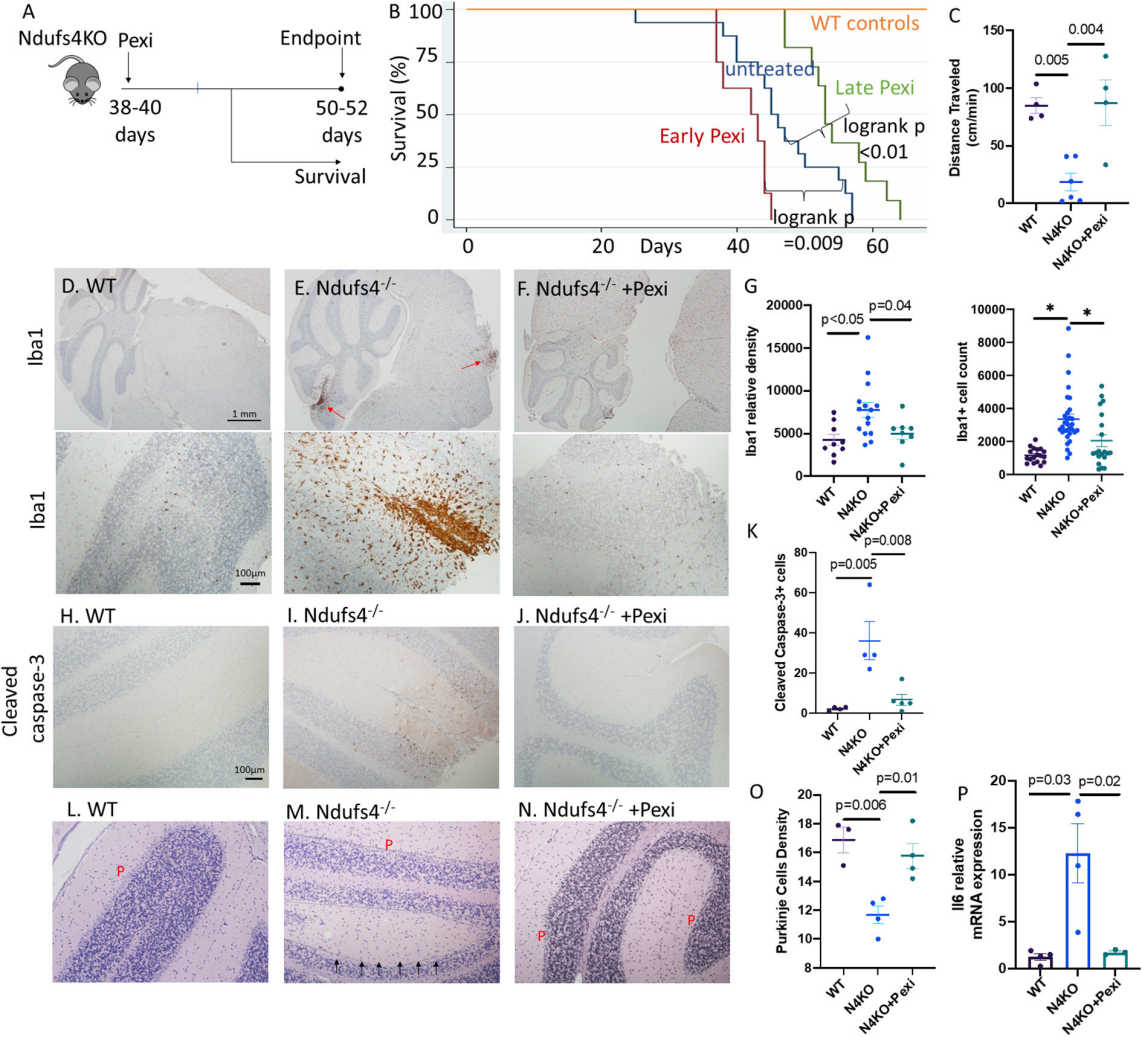
**(A)** Experimental design. **(B)** Survival curves of Ndufs4^−/−^ mice, untreated or saline treated control (*n* = 16), receiving early Pexidartinib (*n* = 8, initiated at D28-37) or late Pexidartinib (*n* = 11 initiated at D38-42). **(C)** Distance traveled (cm/min) in WT and N4KO (LS) mice untreated or treated with Pexidartinib. **(D–F)** Immunohistochemistry of Iba1 (Iow- and high-magnification in upper and lower panels, respectively) and **(G)** Relative densities and cell count of Iba1+ cells from cerebellar cortex and brainstem regions. **(H–J)** Immunohistochemistry of activated/cleaved caspase-3 from mouse brains and **(K)** quantification. **(L–N)** Decreased Purkinje (P) cells (arrow) in saline treated Ndufs4^−/−^ mouse cerebellum **(M)**, which is better preserved in Ndufs4^−/−^ brain from mice treated with late Pexidartinib **(N)** and **(O)** quantification. **(P)** Relative mRNA expression (normalized to 18S) of IL6 from brain of WT, Ndufs4KO, and Pexidartinib treated Ndufs4KO mice (*n* = 3–4 per group). Significance tests by ANOVA followed by Sidak *post-hoc* analysis.

To elucidate the role of microglia, we applied a widely used mouse model of Leigh syndrome with germline homozygous deletion of exon 2 of mitochondrial complex I subunit Ndufs4 (Ndufs4^−/−^). These mice develop severe encephalomyopathy resembling Leigh syndrome (Kruse et al., 2008), characterized by growth retardation, lethargy, loss of motor skill (ataxia), blindness, hypothermia, slowed breathing and death at ~50 days, due to lesions in dorsal brainstem, olfactory bulb and cerebellum, similar to the lesions found in our LS cases. We treated the mice with 1 mg/kg/day of Pexidartinib (Pexi) (Figure 3A), an FDA-approved drug for tenosynovial giant cell tumor. Pexidartinib is a tyrosine kinase inhibitor that also inhibits CSF-1 receptor and is known to ablate microglia from CNS (Elmore et al., 2014). Early Pexi treatment initiated soon after weaning significantly decreased lifespan of LS mice, suggesting a harmful effect of early microglial ablation. However, late Pexi treatment initiated after day 38 (approximately the onset time of neurological symptoms at around 40-day-of-age) significantly extended lifespan (Figure 3B) and ameliorated neurological symptoms, as shown by increased activity measured by average distance traveled (Figure 3C), and much less severe rotational/ataxic movement (Supplementary Videos 1, 2). Neuropathological examinations showed successful reduction of microglia upon late Pexi treatment (*p* = 0.04, Figures 3D–G), with decreased apoptotic cells in the cerebellum and brainstem regions, indicated by significantly decreased cleaved-caspase-3 positive cells by IHC (Figures 3H–K), leading to better-preserved Purkinje neurons (*p* = 0.01, Figures 3L–O). In addition to increased microglial activation in the cerebellum/brainstem of LS mice, the proinflammatory cytokine IL-6 known to be released from activated microglia was significantly increased, and this was suppressed by late Pexi treatment (Figure 3P).

**Figure 4:**
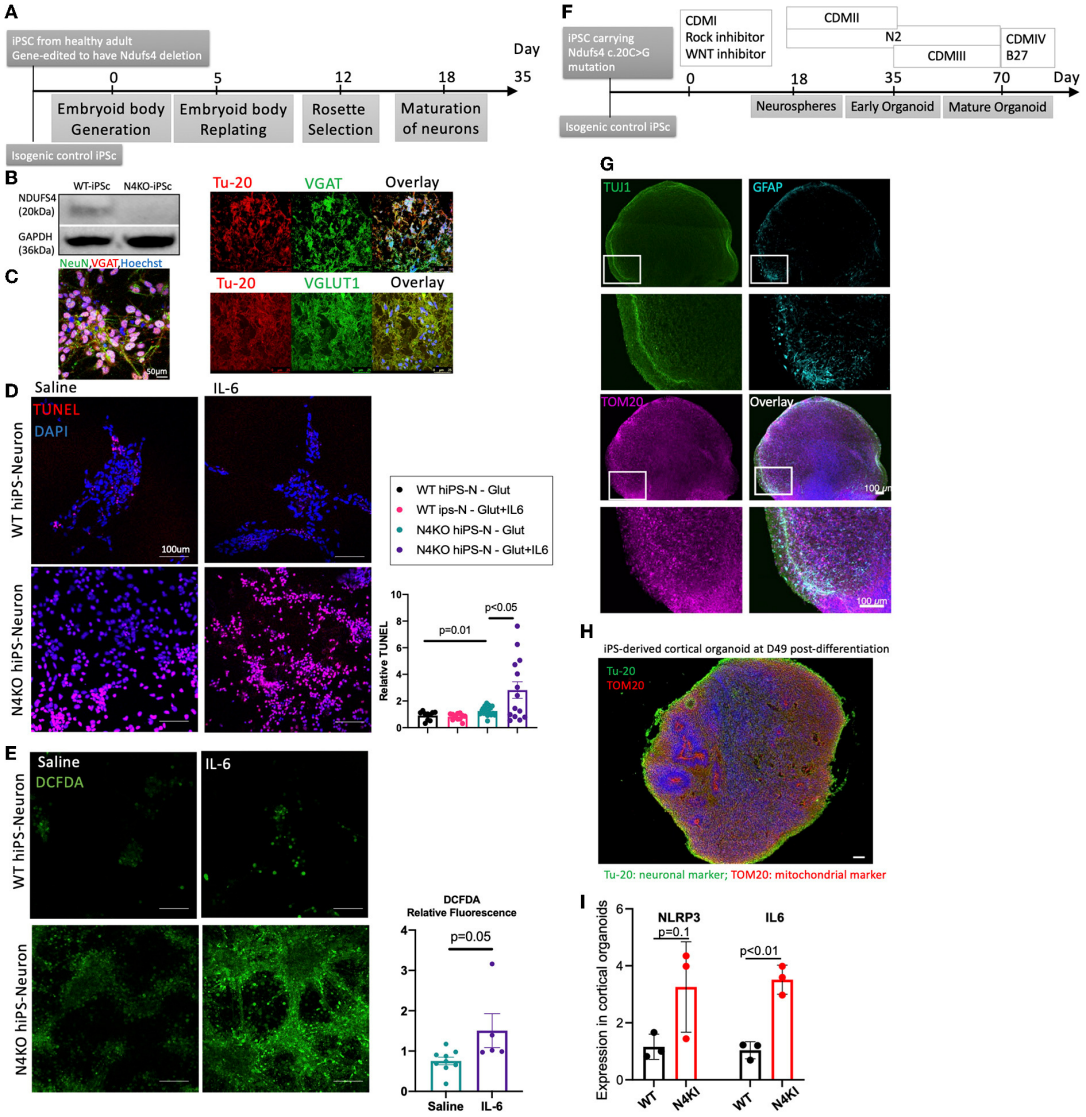
**(A)** Steps to generate iPS-derived neurons. **(B)** Immunoblotting of Ndufs4 in WT-hiPS and Ndufs4KO (N4KO)-hiPS. **(C)** Characterization of human iPS-Ns by immunofluorescence for neuronal nuclear protein (NeuN), pan-neuronal marker b-Tubulin (Tu-20) showing mixed neurons positive for vGAT (vesicular GABA transporter) or vGlut1 (vesicular glutamate transporter). **(D)** TUNEL assay (red) counterstained with DAPI (blue) in WT- and N4KO iPS-neurons treated with glutamate (30 μm), IL-6 (50 μm). **(E)** DCFDA live staining of N4KO iPS-Neurons treated with Saline or IL-6. The significance is calculated by ANOVA Sidak *post-hoc* analysis or *t*-tests. **(F)** Brief protocol to generate iPS-derived cortical organoid. **(G, H)** Representative image of a cortical organoid at day 49 post-differentiation, showing Tuj1 (class III beta-tubulin) for neurons, GFAP (glial fibrillary protein) for astrocytes, TOM-20 staining for mitochondria and Tu-20 staining for neurons. **(I)** Normalized expression of mRNA of NLRP3 and IL6 in cortical organoids at day 80 derived from iPSCs in which we knocked in the mutation c.20C>G in the NDUFS4 gene (N4KI, in red) presented as fold-change compared with isogenic control iPSCs (WT); *n* = three technical replicates, mean + standard deviation; significance by *t*-tests.

To increase the translational value of our findings, we modeled LS using human induced pluripotent stem cell (iPSC)-derived neurons (Figure 4A). Using CRISPR/Cas9 system, we generated Ndufs4-deletion in iPSC from a normal healthy adult and differentiated both Ndufs4-deficient and normal isogenic control into mixed neuronal cells (N4KO-Neurons) (Figure 4B). Approximately 35 days post-differentiation, these iPS-neurons express markers of either Glutamatergic (VGLUT1) or GABAergic (VGAT) neurons, both of which colocalized with the pan-neuronal marker beta-3 tubulin (Tu-20) (Figure 4C). Since activated microglia have been shown to secrete various pro-inflammatory cytokines such as IL-1β, IL-6, and TNF-α in various neurodegenerative diseases, we modeled the secretory effect of activated microglia by treating the control- and N4KO-Neurons with 50 nM IL-6 for 24 h, to recapitulate the humoral effect of activated microglia on the adjacent neurons. The susceptibility to glutamatergic excitotoxicity (30 μM for 6 h) was assessed by TUNEL assay. As shown in Figure 4D, IL-6 significantly increased TUNEL positive N4KO-neurons in response to glutamate challenge. Furthermore, live-staining of N4KO-neurons showed significantly increased DCFDA (2',7'-dichlorodihydrofluorescein diacetate) fluorescence upon IL-6 treatment, suggesting higher total cellular ROS levels in N4KO-neurons in response to IL-6 (Figure 4E).

To investigate the effect of NDUFS4 mutation found in some LS patients, such as NDUFS4 c.20C>G mutation, we generated NDUFS4-mutant by introducing the knock-in c.20C>G mutation in the NDUFS4 on both alleles of normal iPS to create Ndufs4-mutant iPS. Using the mutant iPSC, we differentiated into Ndufs4-mutant brain cortical organoids (Figure 4F). A representative image of these organoids shows a mixture of neurons (Tu-20 or TUJ1 positive cells) and other cells such as astrocytes (marked by GFAP staining) in an organoid context (Figures 4G, H). We did not detect Iba1+ cells in these organoids. To evaluate whether human Ndufs4 mutation can lead to activation of inflammatory pathways, we performed quantitative PCR of the whole organoids at ~80 days post-differentiation. These gene expression analyses revealed ~2–4-fold increase in both NLRP3 (*p* = 0.1) and IL-6 (*p* < 0.01) in Ndufs4-mutant brain organoids, compared with organoids from isogenic WT controls (Figure 4I), suggesting that the human mutation in Ndufs4 mitochondrial complex I subunit is sufficient to induce pro-inflammatory pathways.

## Discussion

In this study we showed novel evidence of microgliosis and features of activated microglia in multiple regions of brains in three pediatric patients died of Leigh syndrome. We further examined the role of microglia in the disease progression and potential treatment of Leigh syndrome using a multipronged approach. In three LS patients examined in the current study, cerebellum and brainstem were severely affected, consistent with the reported lesion topography that brainstem is the most affected structure in LS (Lake et al., 2015), followed by basal ganglia (particularly putamen and substantia nigra), cerebellum (particularly dentate nuclei), thalamus and spinal cord (Cavanagh and Harding, 1994; Rahman et al., 1996). The histopathologic findings were characterized by extensive vacuolation of neuropils, suggestive of degeneration of neuronal processes in the neuropil, although extensive cavitation resembling spongiosis were not obvious in our cases (Wells and Wells, 1989). Capillary/vascular proliferation and thickening were also found in several regions of the brains (Lewis, 1965). There were patchy areas of gliosis, characterized by proliferation and enlarged glial cells, including astrocytes and microglia (van Erven et al., 1987; Cavanagh and Harding, 1994).

Since the current study focus on microglia, we applied IHC for Iba1 to mark microglia. In various parts of postmortem brain from all three LS cases, there was a marked increase of microglial cells (Figure 2). The increased microglia, however, were not easily recognized by H&E staining (Figures 1A–D, 2C, F) and were likely underappreciated in routine neuropathological examinations. Many of the Iba1-positive microglia were enlarged, consistent with activated microglia resembling phagocytic cells (Boche et al., 2013; Hovens et al., 2014). Microglial activation is known to transition from having small, round soma and multiple branching processes to an enlarged ameboid state facilitating the migration of these cells to the site of injury. Acting as resident macrophages of the central nervous system, activated microglia maintain homeostasis by participating in phagocytosis, particularly in the diseased state such as in LS brains. Our novel findings of activated microglia (resembling phagocytic cell) in direct contact with Purkinje neurons (Figure 2E, inset) and the associated loss of Purkinje (Figure 2C) suggest that neuronophagia may play a critical role in the decline of neurons in the affected regions. The phenomenon of neuronophagia is one of the characteristic features of viral or autoimmune encephalitis, and it has not been reported in mitochondrial encephalopathy. To the best of our knowledge, the current study is the first to suggest neuronophagia– cellular-mediated immunity by microglia, in Leigh syndrome encephalopathy.

To examine the role of microglia in LS encephalopathy, we used LS mouse model with Ndufs4^−/−^, which developed multiple brain lesions, particularly in the brainstem and cerebellum, associated with increase in microglia (Quintana et al., 2010; Yoon et al., 2022). We showed that partial ablation of microglia by Pexidartinib may confer protective effect and extend lifespan of Ndufs4^−/−^ mice, but only when it was administered approximately at the onset of neurological symptoms during which activated microglia has been documented (Quintana et al., 2010; Aguilar et al., 2022; Yoon et al., 2022) (late Pexi, Figure 3). A plausible explanation for the detrimental effects of early Pexi in depleting microglia is that microglia play critical physiological roles in phagocytosis of cellular debris, secretion of signaling molecules (e.g., neurotrophins) and essential homeostasis during CNS development, maturation and in diseased state (Anderson and Vetter, 2019). Indeed, microglial activation has been referred to as a “double-edged sword” as it can have both protective or neurotoxic effects on CNS depending on the timing, brain region and the model studied (Prinz et al., 2011; Colonna and Butovsky, 2017). Activated microglia respond to pathogenic insults by cellular-mediated immunity, such as phagocytic function, as our results suggest (Figure 2E, inset), and humoral immunity mediated by cytokines such as IL-6 (Figure 3P). Therefore, the beneficial effect of microglial ablation during the symptom onset in LS is likely attributable to reduction of the pro-inflammatory cytokines and decreased in cellular phagocytosis. However, a recent study using a double knockout mouse line with combined IL-6 and NDUFS4 deficiencies have shown IL-6 deficiency partially improved breathing abnormalities and gliosis but did not extend the lifespan or rescue motor decline in Ndufs4 KO mice(Aguilar et al., 2022).

We examined the effect of Ndufs4-deficiency on iPS-neuron and brain organoid to model human Leigh syndrome encephalopathy. Our data showed that Ndufs4-deficient iPSC neurons were more susceptible to glutamate-induced neuronal excitotoxicity and had increased oxidative stress, and this was further potentiated by exposure to IL-6, a cytokine secreted by microglia to mimic the humoral effect of increased IL6, as found in LS mouse brain and in our Ndufs4-mutant brain cortical organoids. Indeed, increased inflammatory mediators such as IL-6 and NLRP3 inflammasome in Ndufs4-deficient brain organoids suggest that mitochondrial defect due to respiratory complex I mutation is sufficient to induce pro-inflammatory pathways. This is consistent with a mitochondrial-inflammatory link, such as through damage-associated molecular patterns (DAMPs) and inflammasome (Marchi et al., 2022), and this can in turn further aggravates mitochondrial dysfunction.

Human iPSCs have been increasingly used to study human diseases, as it may facilitate clinical translation and may achieve the goal of personalized medicine, such as using mutant iPSC derived from patients to test drug efficacy and toxicity. One limitation of this approach, as with any *in vitro* cell experiment, is the uncertainty of dosage and duration of stressors (e.g., glutamate and IL6 challenge in this study) to recapitulate the actual physiological or pathological scenario. Another limitation is the inadequate maturity of iPSC-derived neurons, since *in vitro* maturation will not match the *in-utero* growth and development, including the degree of mitochondrial maturation (Dai et al., 2017). However, because LS is a disease of very early onset (the age of infant to toddler), iPSC approach has proven to be valuable to model LS (Inak et al., 2021; Yoon et al., 2022). The recent development of iPSC-derived brain organoids has enabled complex modeling of human diseases, such as those involving interactions of multiple cell types, as we reported here to show increased pro-inflammatory pathways in LS brain organoid.

### Clinical implications

Our findings of LS human brain pathology, Ndufs4-deficient mouse and iPSC-neurons and brain organoid models of LS suggest a critical role of activated microglia in the progression of LS encephalopathy. Our findings suggest that not only there are increased numbers and activation of microglia in Leigh syndrome patients, but LS neurons are also more susceptible to proinflammatory cytokines like IL6. Since microglia are essential to support neuronal function, a long-term immunosuppression with Pexidartinib is not recommended. However, our data support a potential therapeutic benefit of microglial inhibition during the flare-up of neuroinflammation, coinciding with activated microgliosis. This is also supported by previous observations in which activated microglia were absent in early-stage Ndufs4^−/−^ mice but became abundant in parallel with disease progression (Quintana et al., 2010). Another explanation is that microglial activation could be protective during early phase (to clear hazardous signals) but excessive pro-inflammatory state at a later phase could be harmful, as seen in AD trajectory (Fan et al., 2017). Delayed short-term ablation of microglia has been also proven beneficial in experimental traumatic brain injury where repopulated microglia did not have the same signaling profile as post injury microglia (Henry et al., 2020). Our findings also suggest that the beneficial effects of Rapamycin in LS mice and patients may be attributable, at least in part, to its immunosuppression effect, given that neuroinflammation has been recently suggested to play a causal role in the pathogenesis of LS (Johnson et al., 2013, 2019; Stokes et al., 2022). Future clinical research will be valuable to identify the biomarkers of neuroinflammation in LS. New imaging techniques such as hyperintensities by MRI and mitochondrial translocator protein (TSPO) imaging by Positron Emission Tomography scanning may provide valuable information about neuroinflammation. Examination of CSF proteins for markers of microglia activation such as MCP-1 is another approach to evaluate neuroinflammation. Implementing these diagnostic approaches have led to detection of microglial activation before any other pathologies are apparent, as reported in AD (Fan et al., 2017), and may be helpful to guide the therapeutic use of microglial ablation in LS patients. Although our iPSC-neurons and brain organoid finding have some limitations as discussed above, we believe that integrating LS mouse model, Ndufs4 mutant iPS-neurons and brain organoid, together with validation using postmortem brain from LS patients, will increase the translational impact of our work. Future studies should include using Leigh syndrome patient-specific iPS cell-derived neurons, microglia and brain organoid, with cell-type specific manipulation to elucidate the interacting mechanisms of microglia and neurons in the context of mitochondrial disease. In summary, our findings provide new insights into the role of microglial-related neuroinflammation in Leigh syndrome and support a potential clinical use of the FDA-approved drug Pexidartinib, under the guidance of biomarkers of neuroinflammation.

## Methods

### Post-mortem neuropathological examinations

All de-identified formalin-fixed brain tissues from LS cases (Table 1) and controls were obtained from the Iowa Neuroscience Institute Neurobank Core, University of Iowa Health and Clinic Pathology Archives and NIH NeuroBioBank. All tissues were paraffin embedded and had the standard 4 μm sections and hematoxylin and eosin (H&E) stain. Control brain sections were obtained from autopsy cases without neurological diseases, including a 26-year-old man and a 23-year-old woman, both died of hanging suicide. The third control was from a 39-year-old man. All post-mortem examinations were performed within 24 h of mortality by neuropathologists.

### Quantitative analysis of pathological images

Semi-quantitative analyses of immunohistochemical staining were performed using NIH Image J, as previously reported (Crowe and Yue, 2019). Briefly, using color deconvolution and threshold adjustment, we removed background noise while obtaining maximal coverage of the DAB staining. The Iba1+ signal areas and total signals were measured for 10 intermediate power images taken using 10 × objective lens (with a coverage area of ~0.87 mm^2^). Arbitrary units were used to compare the Iba-1+ cellular areas and densities of Iba1+ cells among different images (Figures 2, 3). The Iba1 densities are calculated by total signals of Iba1 normalized to the total area of the examined intermediate power fields. The numbers of cleaved caspase 3-positive cells were quantified for 10 high power fields. The density of Purkinje cells was quantified in multiple high-power images and presented as arbitrary unit (with an estimate conversion factor of × 1.257 to obtain an estimated number of Purkinje cells /mm).

### Animal models and experimental design

Animal experiments were approved by the Institutional Animal Care and Use Committee (IACUC) at the University of Iowa. Germline Ndufs4^−/−^ mice were obtained from the University of Washington. All mice were on the C57/BL6/J background and were fed the regular diet from Harlan Teklad. Ndufs4^−/−^ mice were injected daily with 1 mg/kg of Pexidartinib either soon after weaning (early Pexi) or from days 38 to 40 (late Pexi) right before the onset of neurological symptoms. Cross sectional studies of saline or late Pexi treated mice were performed around days 45–48 days.

### Generation of Ndufs4 knock-out iPS cells using CRISPR/Cas9 method

Normal iPS cells generated from a healthy Caucasian male (ATCC-1026) were cultured to 70–80% confluence in 6-cm dishes. Cells were transfected with NF4SgRNA (5'-ATATAAAAACTAGAAAAGTC-3' and 5'-TCGGAACCCTGGAAACGGAA-3') and 5 μl of Lipofectamine 2000 (Invitrogen). Forty-eight hours after transfection, the cells were treated with the appropriate drug and selection was carried out for ~7 days. The cells were then dissociated to single cells. After cells were counted, they were diluted serially in TesR medium to a final concentration of 5 cells per 1-well of 96-well plates and expanded for 2–3 weeks. Visible colonies were picked and reseeded in new wells for monolayer growth. Positive candidates were validated by western blotting and immunostaining. Additionally, to exclude potential off-target effects of CRISPR/Cas9 we performed Sanger sequencing of a few possible target genes predicted by IDT software CRISPR-Cas9 guide RNA design checker. Briefly, regions covering potential off-target sites were amplified using standard PCR. PCR products were identified *via* agarose gel and submitted for Sanger sequencing. We did not find any evidence of off-target mutation at these loci and there are no obvious off-target effects affecting the morphology, growth and other microscopic features.

### Maintenance of human iPS cells and differentiation of mixed neurons

Human iPS cells were obtained from ATCC (1026), which were derived from a healthy adult. The iPS cells cultured on Matrigel-coated plates (Corning, Life Sciences) and fed with a mixture of SFM and mTeSR+ at a ratio of 75:25%. For the generation of neural progenitor cells (NPCs), embryonic body (EB) protocol using STEMdiff neural induction medium and SMADi (STEMdiff™ SMADi Neural Induction Kit, Catalog#05835) was implemented. Briefly, aggrewell^TM^800 plates were prepared for experiments by pre-treatment of the wells with anti-adherence rinsing solution. Subsequently, each well was seeded with 3^*^10^6^ cells of single-cell suspension. Uniform EBs were observed in the aggrewell^TM^800 plates on day 1, ~75% of the medium was replaced with fresh media every day for 4 days. EBs were harvested and replated at day 5 and medium was fully changed each day for 6 days. The percentage of cells induced to a neuronal fate was calculated on Day 8 based on the number of EBs with more than 50% neural rosettes divided by total number of EBs, and it was found to be ~90%. On Day 12 neural rosettes were selected and replated, and full medium changes were performed daily, using STEMdiff^TM^ Neural Induction medium plus SMADi for another 5 days and the NPCs were passaged on Day 18. Neurons were generated from human iPS-NPCs using the STEMdiff^TM^ Neuron Differentiation kit (STEMCELL, catalog #08500) and STEMdiff^TM^ Neuron Maturation kit (STEMCELL, catalog #08510). Briefly, for neuronal differentiation we used a poly-L-ornithine/laminin coating and complete STEMdiff^TM^ Neuron differentiation medium. Neuronal precursors were seeded at a density of 3^*^10^4^ cells/cm^2^ and neurons were allowed to mature for at least 1 week in STEMdiff^TM^ Neuron Maturation medium or Brainphys^TM^ Neuronal medium (STEMCELL, catalog #05790) supplemented with Neurocult^TM^ SM1 Neuronal supplement (STEMCELL, catalog #05711), N2 supplement-A (STEMCELL, catalog #07152), recombinant human brain derived neurotrophic factor, recombinant human glial-derived neurotrophic factor, dibutyryl cAMP, and ascorbic acid.

### Generation of Ndufs4-mutant iPS-derived brain organoids

To model encephalopathy in patients with NDUFS4 c.20C>G mutation, we generated NDUFS4-mutant by introducing the knock-in mutation c.20C>G in the gene NDUFS4 on both alleles of control normal iPSCs. Cortical organoids were generated using our previously published protocol (Le et al., 2021). Briefly, dissociated iPSCs were seeded in CDMI (Glasgow-MEM, 20% knockout serum replacement, 0.1 mM MEM non-essential amino acid solution, 1 mM Sodium Pyruvate, 0.1 mM 2-mercaptethanol, 100 U/mL and 100 μg/mL Penicillin and streptomycin) supplemented with 20 μM ROCK inhibitor, 3 μM WNT-catenin inhibitor (IWR1), and 5 μM SB431542 in 96-well v-bottom plate. On day 1, round cell aggregates (neurospheres) were observed. On day 3, dead cells were detached by tapping on the side of plate and cells were fed with CDMI supplemented with 20 μM ROCK inhibitor, 3 μM IWR1, and 5 μM SB431542. On day 6, medium was removed partially without touching the bottom of the well and cells were fed with CDMI supplemented with 3 μM IWR1 and 5 μM SB431542. This step was repeated every 3 days until day 18. On day 18 neurospheres were transferred to another plate and fed with CDMII (DMEM/F12, 2 mM Glutamax, 1% N-2 supplement, 1% Chemically Defined Lipid Concentrate, 100 U/mL and 100 μg/mL Penicillin and streptomycin) and placed on an orbital shaker inside tissue culture incubator. Media were changed every 3 days until day 35. On day 35 neurospheres were fed with CDMIII (DMEM/F12, 2 mM Glutamax, 1% N2 supplement, 1% Chemically Defined Lipid Concentrate, 100 U/mL and 100 μg/mL Penicillin and streptomycin, 10% Fetal Bovine Serum, 5 μg/mL Heparin, 1% Matrigel) and medium change were performed every 3–5 days depending on the rate of growth. On day 70 CDMIV (DMEM/F12, 2 mM Glutamax, 1% N2 supplement, 1% Chemically Defined Lipid Concentrate, 100 U/mL and 100 μg/mL Penicillin and streptomycin, 10% Fetal Bovine Serum, 5 μg/mL Heparin, 2% Matrigel, 2% B-27 supplement with vitamin A) were used, and medium were changed every 3–5 days.

### Immunostaining studies

iPS-Neurons were plated on glass-bottom dishes. For live staining, the culture medium in the dish was exchanged with prewarmed (37°C) culture medium containing DCFDA (5 μM), incubated for 20 min, then counterstained with Hoechst 33342 in new medium. TUNEL assay was conducted using *In Situ* Cell Death Detection Kit (Roche, 12156792910). For immunostaining, iPS-N cells were fixed in 4% paraformaldehyde, blocked, and incubated in primary antibodies, VGluT1 (Sigma-Aldrich, ZRB2374) and VGAT (Sigma-Aldrich, AMAB91043) overnight. They were then probed with secondary antibody and counterstained with DAPI. Images were acquired using a Leica SP8 confocal microscope.

## Data availability statement

The original contributions presented in the study are included in the article/Supplementary material, further inquiries can be directed to the corresponding author.

## Ethics statement

The animal study was reviewed and approved by Institutional Animal Care and Use Committee (IACUC) at the University of Iowa.

## Author contributions

ND and D-FD performed experiments, data analysis, and manuscript writing. ML and SL performed experiments. MH did neuropathological examinations. D-FD developed the concept and design of the study. AP designed part of the study and provided critical revision of the manuscript. All authors contributed to the article and approved the submitted version.

## References

[B1] Aguilar K. Comes G. Canal C. Quintana A. Sanz E. Hidalgo J. (2022). Microglial response promotes neurodegeneration in the Ndufs4 KO mouse model of Leigh syndrome. Glia. 70, 2032–2044. 10.1002/glia.24234 35770802 PMC9544686

[B2] Anderson S. R. Vetter M. L. (2019). Developmental roles of microglia: a window into mechanisms of disease. Dev. Dyn. 248, 98–117. 10.1002/dvdy.1 30444278 PMC6328295

[B3] Boche D. Perry V. H. Nicoll A. R. (2013). Review: activation patterns of microglia and their identification in the human brain. Neuropathol. Appl. Neurobiol. 39, 3–18. 10.1111/nan.12011 23252647

[B4] Cavanagh J. B. Harding B. N. (1994). Pathogenic factors underlying the lesions in Leigh's disease. Tissue responses to cellular energy deprivation and their clinico-pathological consequences. Brain 117, 1357–1376. 10.1093/brain/117.6.1357 7820572

[B5] Colonna M. Butovsky O. (2017). Microglia function in the central nervous system during health and neurodegeneration. Annu. Rev. Immunol. 35, 441–468. 10.1146/annurev-immunol-051116-052358 28226226 PMC8167938

[B6] Crowe A. R. Yue W. (2019). Semi-quantitative determination of protein expression using immunohistochemistry staining and analysis: an integrated protocol. Bio Protoc. 9. 10.21769/BioProtoc.3465 31867411 PMC6924920

[B7] Dai D.-F. Danoviz M. E. Wiczer B. Laflamme M. A. Tian R. (2017). Mitochondrial maturation in human pluripotent stem cell derived cardiomyocytes. Stem Cells Int. 2017, 5153625. 10.1155/2017/5153625 28421116 PMC5380852

[B8] Davalos D. Grutzendler J. Yang G. Kim J. V. Zuo Y. Jung S.. (2005). ATP mediates rapid microglial response to local brain injury *in vivo*. Nat. Neurosci. 8, 752–758. 10.1038/nn1472 15895084

[B9] Elmore M. R. P. Najafi A. R. Koike M. A. Dagher N. N. Spangenberg E. E. Rice R. A.. (2014). Colony-stimulating factor 1 receptor signaling is necessary for microglia viability, unmasking a microglia progenitor cell in the adult brain. Neuron 82, 380–397. 10.1016/j.neuron.2014.02.040 24742461 PMC4161285

[B10] Fan Z. Brooks D. J. Okello A. Edison P. (2017). An early and late peak in microglial activation in Alzheimer's disease trajectory. Brain 140, 792–803. 10.1093/brain/aww349 28122877 PMC5837520

[B11] Henry R. J. Ritzel R. M. Barrett J. P. Doran S. J. Jiao Y. Leach J. B.. (2020). Microglial depletion with CSF1R inhibitor during chronic phase of experimental traumatic brain injury reduces neurodegeneration and neurological deficits. J. Neurosci. 40, 2960–2974. 10.1523/JNEUROSCI.2402-19.2020 32094203 PMC7117897

[B12] Hopperton K. E. Mohammad D. Trépanier M. O. Giuliano V. Bazinet R. P. (2018). Markers of microglia in post-mortem brain samples from patients with Alzheimer's disease: a systematic review. Mol. Psychiatry 23, 177–198. 10.1038/mp.2017.246 29230021 PMC5794890

[B13] Hovens I. B. Nyakas C. Schoemaker R. G. (2014). A novel method for evaluating microglial activation using ionized calcium-binding adaptor protein-1 staining: cell body to cell size ratio. Neuroimmunol. Neuroinflamm. 1, 82–88. 10.4103/2347-8659.139719

[B14] Inak G. Rybak-Wolf A. Lisowski P. Pentimalli T. M. Jüttner R. GlaŽar P.. (2021). Defective metabolic programming impairs early neuronal morphogenesis in neural cultures and an organoid model of Leigh syndrome. Nat. Commun. 12, 1929. 10.1038/s41467-021-22117-z 33771987 PMC7997884

[B15] Johnson S. C. Martinez F. Bitto A. Gonzalez B. Tazaerslan C. Cohen C.. (2019). mTOR inhibitors may benefit kidney transplant recipients with mitochondrial diseases. Kidney Int. 95, 455–466. 10.1016/j.kint.2018.08.038 30471880 PMC6389356

[B16] Johnson S. C. Yanos M. E. Kayser E.-B. Quintana A. Sangesland M. Castanza A.. (2013). mTOR inhibition alleviates mitochondrial disease in a mouse model of Leigh syndrome. Science 342, 1524–1528. 10.1126/science.1244360 24231806 PMC4055856

[B17] Kruse S. E. Watt W. C. Marcinek D. J. Kapur R. P. Schenkman K. A. Palmiter R. D. (2008). Mice with mitochondrial complex I deficiency develop a fatal encephalomyopathy. Cell Metab. 7, 312–320. 10.1016/j.cmet.2008.02.004 18396137 PMC2593686

[B18] Lake N. J. Bird M. J. Isohanni P. Paetau A. (2015). Leigh syndrome: neuropathology and pathogenesis. J. Neuropathol. Exp. Neurol. 74, 482–492. 10.1097/NEN.0000000000000195 25978847

[B19] Le S. Petersilie L. Inak G. Menacho-Pando C. Kafitz K. W. Rybak-Wolf A.. (2021). Generation of human brain organoids for mitochondrial disease modeling. J. Vis. Exp. e62756. 10.3791/62756 34223837

[B20] Lewis A. J. (1965). Infantile subacute necrotizing encephalopathy. Can. Med. Assoc. J. 93, 878–881. 5829183 PMC1928961

[B21] Marchi S. Guilbaud E. Tait S. W. G. Yamazaki T. Galluzzi L. (2022). Mitochondrial control of inflammation. Nat. Rev. Immunol. 10.1038/s41577-022-00760-x 35879417 PMC9310369

[B22] Nimmerjahn A. Kirchhoff F. Helmchen F. (2005). Resting microglial cells are highly dynamic surveillants of brain parenchyma *in vivo*. Science 308, 1314–1318. 10.1126/science.1110647 15831717

[B23] Prinz M. Priller J. Sisodia S. S. Ransohoff R. M. (2011). Heterogeneity of CNS myeloid cells and their roles in neurodegeneration. Nat. Neurosci. 14, 1227–1235. 10.1038/nn.2923 21952260

[B24] Quintana A. Kruse S. E. Kapur R. P. Sanz E. Palmiter R. D. (2010). Complex I deficiency due to loss of Ndufs4 in the brain results in progressive encephalopathy resembling Leigh syndrome. Proc. Natl. Acad. Sci. U. S. A. 107, 10996–11001. 10.1073/pnas.1006214107 20534480 PMC2890717

[B25] Rahman S. Blok R. B. Dahl H. H. Danks D. M. Kirby D. M. Chow C. W.. (1996). Leigh syndrome: clinical features and biochemical and DNA abnormalities. Ann. Neurol. 39, 343–351. 10.1002/ana.410390311 8602753

[B26] Ruhoy I. S. Saneto R. P. (2014). The genetics of Leigh syndrome and its implications for clinical practice and risk management. Appl. Clin. Genet. 7, 221–234. 10.2147/TACG.S46176 25419155 PMC4235479

[B27] Schafer D. P. Lehrman E. K. Kautzman A. G. Koyama R. Mardinly A. R. Yamasaki R.. (2012). Microglia sculpt postnatal neural circuits in an activity and complement-dependent manner. Neuron 74, 691–705. 10.1016/j.neuron.2012.03.026 22632727 PMC3528177

[B28] Stokes J. C. Bornstein R. L. James K. Park K. Y. Spencer K. A. Vo K.. (2022). Leukocytes mediate disease pathogenesis in the Ndufs4(KO) mouse model of Leigh syndrome. JCI Insight. 7, e156522. 10.1172/jci.insight.156522 35050903 PMC8983133

[B29] Ueno M. Fujita Y. Tanaka T. Nakamura Y. Kikuta J. Ishii M.. (2013). Layer V cortical neurons require microglial support for survival during postnatal development. Nat. Neurosci. 16, 543–551. 10.1038/nn.3358 23525041

[B30] van Erven P. M. Cillessen J. P. Eekhoff E. M. Gabreëls F. J. Doesburg W. H. Lemmens W. A.. (1987). Leigh syndrome, a mitochondrial encephalo(myo)pathy: a review of the literature. Clin. Neurol. Neurosurg. 89, 217–230. 10.1016/S0303-8467(87)80020-3 3319345

[B31] Wells G. A. Wells M. (1989). Neuropil vacuolation in brain: a reproducible histological processing artefact. J. Comp. Pathol. 101, 355–362. 10.1016/0021-9975(89)90018-2 2691536

[B32] Yoon J.-Y. Daneshgar N. Chu Y. Chen B. Hefti M. Vikram A.. (2022). Metabolic rescue ameliorates mitochondrial encephalo-cardiomyopathy in murine and human iPSC models of Leigh syndrome. Clin. Transl. Med. 12, e954. 10.1002/ctm2.954 35872650 PMC9309541

